# Targeting CD16A on NK cells and GPC3 in hepatocellular carcinoma: development and functional validation of a therapeutic bispecific antibody

**DOI:** 10.3389/fimmu.2025.1599764

**Published:** 2025-06-12

**Authors:** Liu Chen, Yuankui Zhu, Mingqian Feng, Dianbao Zuo, Guoping Chen, Kangkang Ji

**Affiliations:** ^1^ College of Life Science and Technology, Huazhong Agricultural University, Wuhan, Hubei, China; ^2^ College of Biomedicine and Health, Huazhong Agricultural University, Wuhan, Hubei, China; ^3^ Research Center for Translational Medicine, Hubei Key Laboratory of Wudang Local Chinese Medicine Research, Hubei Provincial Clinical Research Center for Parkinson’s Disease at Xiangyang No.1 People’s Hospital, Hubei University of Medicine, Xiangyang, China; ^4^ Department of Clinical Medical Research, Binhai County People’s Hospital, Binhai Clinical College, Yangzhou University Medical College, Yancheng, Jiangsu, China

**Keywords:** bispecific antibody, NK cell engager, CD16A, glypican-3, hepatocellular carcinoma

## Abstract

**Introduction:**

Advanced hepatocellular carcinoma (HCC) poses significant therapeutic challenges due to chemotherapy resistance and limited efficacy of current targeted therapies. To address this unmet need, we developed a bispecific antibody (BsAb) platform targeting CD16A on natural killer (NK) cells and glypican-3 (GPC3), a tumor-specific antigen overexpressed in 70% of HCC cases.

**Methods:**

High-affinity anti-CD16A single-chain variable fragments (scFvs) were selected via phage display, followed by engineering of Fc-stabilized BsAbs (MA4-hFc(N297A)-CD16A series) to minimize FcγR-mediated toxicity. Functional validation included binding kinetics (ELISA, flow cytometry, and fluorescence co-localization analysis), *in vitro* cytotoxicity assays (luciferase-based killing), and *in vivo* efficacy studies in Huh7 xenograft models. Synergy with sorafenib was assessed using CompuSyn analysis.

**Results:**

The lead candidate, MA4-hFc-CD16AM19, exhibited nanomolar affinity (EC50 < 10 nM for human CD16A) with no murine cross-reactivity. It demonstrated potent, dose-dependent cytotoxicity against GPC3+ HCC lines (HepG2/Huh7/Hep3B, IC50 = 15–35 ng/mL) via NK cell activation, surpassing conventional antibody-dependent cellular cytotoxicity (ADCC). Combined with sorafenib, MA4-hFc-CD16AM19 achieved synergistic tumor suppression (CI=0.41). *In vivo*, BsAb treatment (5 mg/kg) significantly inhibited tumor growth in xenograft models, correlating with enhanced intratumoral NK cell infiltration without toxicity.

**Conclusion:**

This study introduces three innovations: (1) a species-specific CD16A binder overcoming polymorphism limitations, (2) Fc domain engineering (N297A) to optimize stability and safety, and (3) a synergistic combination strategy with sorafenib. The results provide a translatable framework for GPC3+ solid tumor immunotherapy.

## Graphical Abstract

Mechanistic basis of bispecific antibody MA4-hFc-CD16A-mediated tumor cell cytotoxicity.

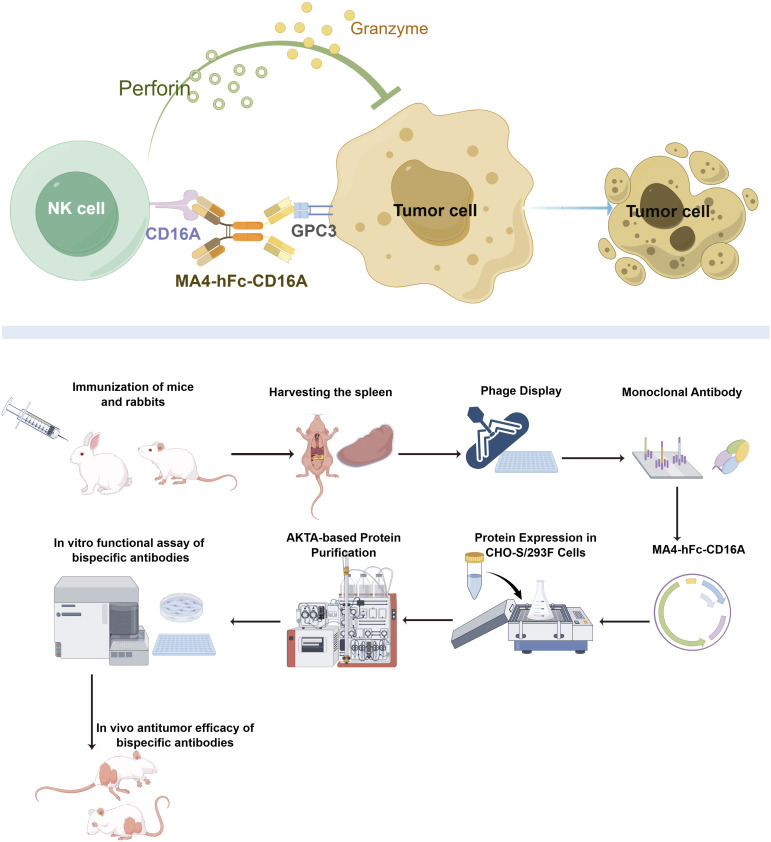

## 1 Introduction

Hepatocellular carcinoma (HCC) continues to rank among the most lethal malignancies globally, exhibiting a five-year survival rate below 20% with particularly limited therapeutic options for advanced-stage patients (1, 2). While tyrosine kinase inhibitors (TKIs) like sorafenib have modestly improved survival outcomes, their clinical utility remains constrained by suboptimal response rates (<30%) and frequent development of drug resistance (3–5). Recent advances in immunotherapy have brought Glypican-3 (GPC3) into focus as a prime therapeutic target - this heparan sulfate proteoglycan demonstrates tumor-specific overexpression in 70% of HCC cases while maintaining near absence in normal liver tissue (6, 7). Preclinical validation of GPC3-targeting immunotoxins has further reinforced its therapeutic potential through demonstrated high specificity and potent cytotoxicity in HCC models (8).

Emerging immunotherapeutic strategies including GPC3-directed CAR-T cells and monoclonal antibodies (e.g., codrituzumab) have shown preclinical promise, yet face clinical translation barriers including inadequate tumor infiltration of effector cells and tumor microenvironment-mediated immunosuppression (8–10). Conventional monoclonal antibodies relying on Fc-mediated antibody-dependent cellular cytotoxicity (ADCC) face additional limitations due to insufficient activation of immune effector cells within HCC’s immunosuppressive milieu (11, 12). This therapeutic gap has spurred interest in natural killer (NK) cell-engaging bispecific antibodies (BsAbs) that leverage CD16A (FcγRIIIa) engagement to circumvent Fc-dependent activation constraints (13, 14). Significantly, CD16A polymorphism-dependent efficacy variations (e.g., 158V/F) and the ability of direct CD16A engagement to amplify NK cell antitumor activity independent of Fc mechanisms highlight the strategic value of this approach (15–17).

While CD16A-targeting BsAbs such as AFM13 (targeting CD30/CD16A) have demonstrated clinical efficacy in hematologic malignancies (18, 19), their application in solid tumors like HCC remains largely unexplored. Current GPC3-based BsAbs exemplified by ERY974 (GPC3/CD3) predominantly employ T cell recruitment strategies, yet confront challenges including cytokine release syndrome (CRS) and T cell exhaustion (20, 21). These limitations underscore the critical unmet need for optimized NK cell-redirecting BsAbs that balance therapeutic efficacy with safety, while maintaining potential for synergistic combination with kinase inhibitors.

Despite remarkable clinical successes of BsAbs in hematological malignancies, as evidenced by blinatumomab (CD19/CD3) (22–26), their adaptation to solid tumors demands meticulous optimization across three key parameters: target antigen selection, antibody structural architecture, and toxicity mitigation strategies (27). To address these challenges, we developed a novel panel of six GPC3/CD16A bispecific antibodies through integrated phage display screening and Fc domain engineering. Our constructs combine high-affinity GPC3-targeting scFv domains (EC50 <10 nM) with diversified CD16A-binding modalities, incorporating N297A glycosylation ablation to eliminate FcγR-mediated off-target effects while preserving structural integrity.

Comprehensive functional validation was conducted through a three-dimensional optimization strategy: (1) Species specificity screening to ensure exclusive human CD16A reactivity for translational relevance; (2) Biophysical characterization assessing Fc engineering (N297A) impacts on thermostability and NK cell recruitment efficiency; (3) Therapeutic synergy evaluation with sorafenib to overcome microenvironment-driven resistance. Utilizing an integrative approach encompassing binding kinetics analysis (flow cytometry), *in vitro* cytotoxicity profiling (luciferase-based killing assays), and *in vivo* efficacy assessment (Huh7 xenograft models), this study establishes a systematic framework for developing next-generation NK-engaging BsAbs against solid tumors.

## 2 Methods

### 2.1 Cell lines and peripheral blood mononuclear cells

Three GPC3-positive cell lines (Huh7, Hep3B, and HepG2) and the GPC3/CD16A-negative A431 cell line were maintained as adherent monolayers in Dulbecco’s modified Eagle’s medium (DMEM, Invitrogen) supplemented with 10% fetal bovine serum (HyClone), 1% L-glutamine, and 1% penicillin-streptomycin (Invitrogen), under 5% CO_2_ at 37°C. CD16A-negative A431 cells (a human epithelial carcinoma line) were transfected with a plasmid encoding full-length CD16A (GenBank accession: NP_000560.7) to establish stable CD16A overexpression. Both parental A431 and transfected A431-CD16A cells were cultured in DMEM. All cell lines were obtained from the National Cancer Institute (NCI) at the National Institutes of Health (NIH) and transduced with a lentiviral vector expressing a luciferase-GFP fusion gene for cytotoxicity assays. Human PBMCs were isolated from whole blood of healthy donors (Wuhan Blood Center) using Ficoll density gradient centrifugation (Stem Cell Technologies).

### 2.2 Design and expression of CD16A recombinant protein

The coding sequence (CDS) of CD16A (FcγRIIIa-158V, 254 amino acids) was synthesized by GenScript based on the NCBI reference sequence NM_000569. Primers were designed to amplify the extracellular domain (ECD) of CD16A, which was fused with a C-terminal His-tag and cloned into a pcDNA3.1 vector. The recombinant plasmid was transiently transfected into HEK 293F cells (ATCC CRL-1573) using the Expi293 expression system (Thermo Fisher). The recombinant protein was purified via Ni-NTA affinity chromatography (Qiagen), with purity verified by SDS-PAGE (Bio-Rad) and concentration determined using a NanoDrop ND-2000 spectrophotometer.

### 2.3 Animal immunization and serum titer analysis

Immunization protocols were as follows: BALB/c female mice (6–8 weeks old, n=5) received a primary immunization with 50 μg CD16A-His protein mixed with a water-soluble adjuvant (BoaoLong), followed by boosters every two weeks (three total) (28). New Zealand white rabbits (n=2) were immunized with 200 μg CD16A-His per animal using the same adjuvant and schedule. Serum samples were collected 1 week post-immunization via tail vein bleeding (mice) or ear artery puncture (rabbits). Serum titers were measured by ELISA (specific polyclonal antibody titers defined as OD450 >1.0 at 1:10^4^ dilution). Spleens were harvested for library construction once target titers were achieved (29).

The experimental procedure for RNA extraction from mouse spleen tissue is performed as follows: First, the spleen is aseptically removed and homogenized in a cryogenic mill with 1 mL of TRIzol reagent to obtain a uniform lysate. Subsequently, 200 μL of chloroform is added, and the mixture is vigorously vortexed for 15 seconds before incubation at room temperature for phase separation. Centrifugation at 12,000 × g (4°C, 15 min) allows collection of the RNA-containing aqueous supernatant. This supernatant is mixed with an equal volume of ice-cold isopropanol, incubated for 10 min at 4°C, and centrifuged again (12,000 × g, 10 min) to pellet RNA. The pellet undergoes two washes with pre-chilled 75% ethanol (8,000 × g, 5 min per wash) to remove impurities. After air-drying residual ethanol, the RNA is dissolved in RNase-free water, with concentration and purity assessed spectrophotometrically (A260/A280 ratio). Critical considerations include maintaining RNase-free conditions, ensuring complete phase separation during chloroform treatment, and avoiding RNA pellet disruption during liquid aspiration steps.

### 2.4 Phage library construction and screening

Total RNA from splenocytes was reverse-transcribed to cDNA, and variable heavy (VH) and light (Vκ) chains were amplified via overlap PCR to generate scFv fragments. These fragments were cloned into the pComb3XSS phage display vector (30). Initial library sizes reached 5 × 10^9^ CFU/mL (murine) and 1.56 × 10^9^ CFU/mL (rabbit). Biopanning was performed using decreasing antigen concentrations (10–1 μg/mL CD16A-His) over four rounds. Six murine and four rabbit-derived monoclonal antibodies (mAbs) were selected for further characterization (29, 31).

### 2.5 *In vitro* characterization of monoclonal antibodies

#### 2.5.1 Protein expression

ScFv fragments fused to a 6×His-FLAG tag (scFv-HF) were expressed in E. coli HB2151 using the pComb3X vector. Colonies were cultured to OD600 = 0.6–0.8, induced with 0.5 mM IPTG at 16°C for 8 h, and lysed with polymyxin B (50 μg/mL). Supernatants were purified via Ni-NTA affinity chromatography, eluted with a 250 mM imidazole gradient, and buffer-exchanged into PBS.

#### 2.5.2 Binding affinity (ELISA)

mCD16/CD16A-His (5 μg/mL) was coated onto plates, blocked with 2% BSA-PBS, and incubated with serially diluted scFv-HF (0.1–300 nM). IL6-HisFlag was used as a labelling control antibody. Bound antibodies were detected using HRP-conjugated anti-FLAG antibody, followed by TMB substrate. EC_50_ values were calculated via four-parameter logistic regression (GraphPad Prism 5).

#### 2.5.3 Western blot

For protein analysis, 10 ng of recombinant CD16A-His protein was mixed with 5× reducing loading buffer and denatured at 95°C for 10 min in a final volume of 20 μL. Samples and pre-stained protein markers were resolved on SDS-PAGE gels through electrophoresis, terminated when the dye front approached the gel bottom. Proteins were transferred onto PVDF membranes at 250 mA for 60 min using wet transfer methodology. Membranes were blocked with 5% non-fat milk in TBST (Tris-buffered saline with 0.1% Tween-20) for 1 h at room temperature to minimize non-specific binding. Primary antibody incubation was performed overnight at 4°C with anti-Flag antibody (1 μg/mL in blocking buffer). Following three 5-min TBST washes, membranes were probed with HRP-conjugated mouse anti-Flag secondary antibody (1:5,000 dilution) for 1 h at room temperature. Four additional TBST washes (5 min each) preceded signal development using enhanced chemiluminescent substrate (ECL), with image acquisition performed via a digital gel documentation system.

#### 2.5.4 Cell binding (flow cytometry)

Cells were harvested with trypsin-EDTA, washed, and resuspended in 5% BSA-PBS. Surface CD16A expression was assessed using 10 μg/mL scFv-HF, detected with APC-conjugated anti-His antibody (BioLegend). For PBMC analysis, FITC-conjugated goat anti-rabbit secondary antibodies (Abclonal) were utilized.

### 2.6 Design and functional validation of bispecific antibodies

#### 2.6.1 Bispecific antibody construction

A tandem scFv format was employed, linking GPC3-scFv-hFc(N297A) to CD16A-scFv (N297A mutation in Fc to eliminate glycosylation and ADCC activity) (32). This architecture follows recent biophysical insights showing that G4S linkers improve conformational flexibility and antigen engagement. Constructs were cloned into CHO-K1 (ATCC CCL-61) and HEK 293F cells via Hind III digestion and selected with puromycin. Following PEI-mediated transfection, genetically modified cells were subjected to puromycin selection (30 μg/mL) in 96-well plates 72 h post-transfection. Surviving monoclonal colonies were expanded and screened for antibody secretion using quantitative sandwich ELISA. High-producing clones exhibiting superior antibody titers were prioritized for scale-up production. Selected stable cell pools were propagated in suspension culture using Erlenmeyer shake flasks under standardized conditions (37°C, 5% CO_2_, 120 rpm). Culture supernatants were harvested at peak productivity phases, clarified by centrifugation (4,000 × g, 20 min), and subjected to Protein A affinity chromatography for IgG purification. Target antibodies were eluted using low-pH buffer (0.1 M glycine-HCl, pH 2.7) followed by immediate neutralization and buffer exchange into PBS via tangential flow filtration.

#### 2.6.2 Binding activity (ELISA)

Microplates were immobilized with 50 μL of recombinant GPC3/CD16A(ECD)-His protein (5 μg/mL in PBS, pH 7.4) through 30-min incubation at 37°C. Following three PBST (PBS containing 0.05% Tween-20) washes, non-specific binding sites were blocked using 2% BSA in PBST for 30 min at 37°C. Serial three-fold dilutions of bispecific antibodies (100 nM starting concentration in PBS) were applied to the plates (50 μL/well) and incubated for 30 min at 37°C. After removing unbound antibodies and performing two PBST washes, HRP-conjugated rabbit anti-human IgG (1:8,000 dilution; Boiron, Shanghai) was added (50 μL/well) for 30 min at 37°C. Following four PBST wash cycles, enzymatic activity was quantified by adding 50 μL TMB substrate (KPL) for 5 min protected from light. The reaction was terminated with 50 μL of 0.5 M H_2_SO_4_, with absorbance measured at 450 nm using a multimode microplate reader (Molecular Devices).

#### 2.6.3 Flow cytometric validation

Adherent cells were enzymatically dissociated using 0.25% trypsin-EDTA (Thermo Fisher Scientific, Waltham, MA), pelleted by centrifugation (300 × g, 5 min), and resuspended in ice-cold PBS supplemented with 5% BSA at a density of 1 × 10^6^ cells/mL. Cells were incubated with primary antibody (10 μg/mL in blocking buffer) for 90 min on ice with gentle agitation. Following two washes with cold PBS, secondary staining was performed using Cy5-conjugated goat anti-human IgG (1:500 dilution; Jackson ImmunoResearch, West Grove, PA) for 45 min under light-protected conditions. Cell suspensions were analyzed on a BD FACS Calibur flow cytometer (BD Biosciences, Franklin Lakes, NJ), with fluorescence signals quantified using CellQuest Pro software.

#### 2.6.4 Fluorescence co-localization analysis

Tumor cells (LG; A431, G1, Huh7) at a density of 5,000 per well were seeded into 24-well plates and subsequently cultured at 37°C with 5% CO_2_ for a period of 24 hours. Thereafter, CY5-labeled CD16AM5 and MA4-hFc-CD16AM5 antibodies (10 µg/ml) were added to the PBMCs corresponding to the tumor cells at a ratio of PBMC:tumor = 5:1. The incubation period was continued for a duration of 24 hours, during which direct microscopic observation was performed. The bright field channel is utilized for the observation of the morphology and distribution of PBMCs. The CY5 channel is employed for the detection of the fluorescence signal of the double antibody on the surface of tumor cells, while the FITC channel is used for the observation of tumor cells.

#### 2.6.5 *In vitro* cytotoxicity

The assessment of cell growth and viability was conducted through the implementation of a firefly luciferase reporter gene assay. All of the cancer cells that were utilized in the experiment were stably infected, and the firefly luciferase reporter gene (ffLuc2) was constitutively expressed. A volume of 200 μL of stably transduced cells was inoculated into a 96-well plate (5 x 10^3^ cells per well). Thereafter, PBMC cells (1 x 10^5^) were added, along with the indicated concentrations of bispecific antibodies (0.01-10,000 ng/mL). Following a 72-hour incubation at 37°C, the culture media were aspirated, and 100 µL of phosphate buffered saline (PBS) was added. This was followed by two cycles of freeze-thaw lysis. The release of luciferase activity was measured to represent cell viability. The *in vitro* assay for measuring cellular toxicity was conducted on three separate occasions, utilizing PBMCs obtained from three distinct donors. Sorafenib (0.0137–10 μM, Solarbio) was tested for synergy using CompuSyn (CI values) (33).

#### 2.6.6 *In vivo* antitumor efficacy

All mice were treated in accordance with the protocol that had been approved by the Animal Care and Use Committee of Huazhong Agricultural University (approval number: HZAUMO-2017-051). The female NOD-SCID mice (8 weeks old) were subcutaneously inoculated with 5 × 10^6^ Huh7 cells. When tumors reached 150 mm³, mice received cyclophosphamide (50 mg/kg, i.p.) to deplete lymphocytes. PBMCs (8 × 10^6^/mouse) and BsAbs (2.5 or 5 mg/kg, i.v.) were administered twice weekly for two weeks. Tumor volume was calculated as (length × width²)/2.

### 2.7 Statistical analysis

Data are expressed as mean ± SEM. All experiments were conducted with three biological replicates. Statistical analysis was performed using one-way ANOVA with Tukey’s *post hoc* test (GraphPad Prism 9.0), with significance defined as p < 0.05.

## 3 Results

### 3.1 Characterization of CD16A monoclonal antibodies

#### 3.1.1 Recombinant protein and antibody affinity

**Figure 1:**
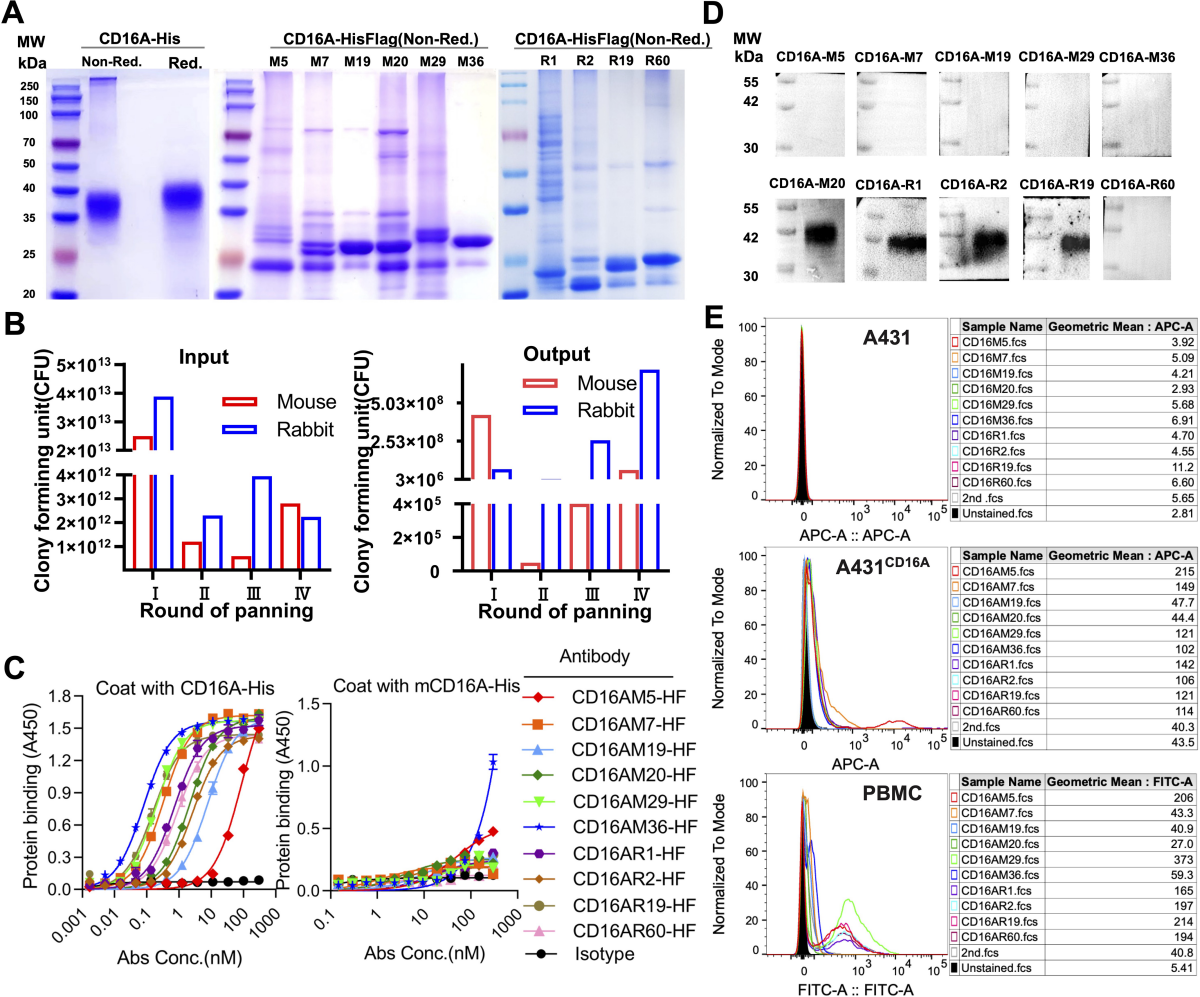
Phage display-derived CD16A monoclonal antibodies exhibit nanomolar affinity and species specificity. **(A)** SDS-PAGE analysis of recombinant CD16A extracellular domain (ECD)-His and CD16A monoclonal antibodies (mAbs). Proteins (5 µg/lane) were separated under non-reducing (Non-Red.) or reducing (Red.) conditions. CD16A(ECD)-His was expressed in HEK293F cells, while CD16A mAbs were produced in HB2151 **(E)** coli and purified via Ni-NTA affinity chromatography. The apparent molecular weight of CD16A(ECD)-His (~40 kDa) exceeds the theoretical value (21 kDa) due to extensive glycosylation at N-linked sites. SDS-PAGE heterogeneity in mAbs (lanes M5-M36) reflects prokaryotic expression challenges, including host-derived impurities (e.g., BL21 endotoxins), variable IPTG induction efficiency across clones, and non-specific protein binding during Ni-NTA elution (imbalanced imidazole concentrations). **(B)** Enrichment profile of phage libraries across sequential biopanning rounds using CD16A-immunized mice. Input reflects total phage quantity per round, while output represents target-bound phage recovered after elution. **(C)** ELISA-based evaluation of CD16A mAb binding affinity and specificity. Plates coated with CD16A-His (5 µg/mL) were incubated with serially diluted mAbs. Binding was detected using HRP-conjugated anti-His antibody. IL6-HisFlag served as a negative control for HisTag specificity. **(D)** Western blot confirmation of CD16A mAb specificity. CD16A-His (10 ng) was resolved by SDS-PAGE, transferred to PVDF membranes, and probed with mAb (1 µg/mL). HRP-conjugated anti-Flag antibody was used for detection. **(E)** Flow cytometric analysis of CD16A mAb binding to A431 (CD16A-negative) and A431-CD16A+ cells. Cells were stained with mAb (90 min, 4°C) and detected via Cy5-labeled anti-His antibody. For PBMCs, a rabbit anti-His primary antibody followed by FITC-conjugated goat anti-rabbit secondary antibody was employed.

CD16A monoclonal antibodies (mAbs) were generated through murine immunization and phage display. The extracellular domain of CD16A (CD16A(ECD)) fused with a His-tag was expressed in HEK 293F cells. SDS-PAGE confirmed high purity of the purified protein (**Figure 1A**). Following immunization of five BALB/c mice and two New Zealand white rabbits with the recombinant protein, phage-displayed antibody libraries were constructed using splenic RNA. Four rounds of biopanning with CD16A-His (**Figure 1B**) yielded six murine-derived and four rabbit-derived CD16A mAbs (scFv-HisFlag), which were expressed and purified in E. coli. SDS-PAGE confirmed bands corresponding to the expected molecular weights (**Figure 1A**). ELISA revealed specific binding of all scFv-HF antibodies to CD16A(ECD)-His (**Figure 1C**), with EC50 values ranging from 0.08 to 60.73 nM (**Table 1**). M29, M36, and R19 exhibited the highest affinities.

**Table 1:** Detection of 10 monoclonal antibody affinities (EC50).

mAb(CD16)	M5	M7	M19	M20	M29	M36	R1	R2	R19	R60
EC50	60.73	0.345	8.14	2.418	0.204	0.086	0.76	2.708	0.172	0.951

Cross-reactivity assays demonstrated binding of all CD16A mAbs to human CD16 but not murine mCD16A (OD450 <0.5), confirming species specificity (**Figure 1C**). Western blot further validated that four antibodies (e.g., CD16AM20 and CD16AR1) recognized linear epitopes of denatured CD16A (**Figure 1D**), while flow cytometry indicated strongest binding of CD16AM29 and CD16AR19 to native CD16A conformations (**Figure 1E**), suggesting distinct epitope preferences. Western blot band stacking at ~40 kDa reflects CD16A(ECD)-His glycosylation heterogeneity. The absence of visible bands for conformational epitope-binding mAbs (M5/M7/M29/M36) in non-denaturing conditions aligns with their inability to recognize linearized antigens (validated in **Figures 1D-E**). Membrane artifacts (stray dots) resulted from transient air bubble entrapment during transfer and incomplete dissolution of blocking reagents. Flow cytometric validation was performed on CD16-negative A431 cells, CD16-positive primary human NK cells (PBMCs), and an engineered A431-CD16A cell line. As expected, none of the mAbs bound to parental A431 cells. In A431-CD16A cells, most mAbs exhibited weak binding except CD16AM19 and CD16AM20, likely due to challenges in re-screening with A431-CD16A cells (**Figure 1E**). Among human PBMCs (where NK cells account for ~15%), the highest binding activities were observed for CD16AM29, CD16AR19, CD16AM5, CD16AR2, CD16AR60, and CD16AR1. The weak CD16A signal in engineered A431-CD16A cells versus PBMCs arises from two factors: (1) Pre-existing selection markers (LG) in A431-LG cells hindered lentiviral transduction efficiency during CD16A overexpression, yielding lower surface expression than endogenous NK cells; (2) Epitope accessibility differences—the MA4-hFc-CD16A series preferentially binds native conformational epitopes that may be partially masked in the artificial overexpression system, whereas PBMC-derived NK cells present naturally processed CD16A conformers.

### 3.2 Functional validation of bispecific antibodies

#### 3.2.1 Bispecific antibody design and binding activity

**Figure 2:**
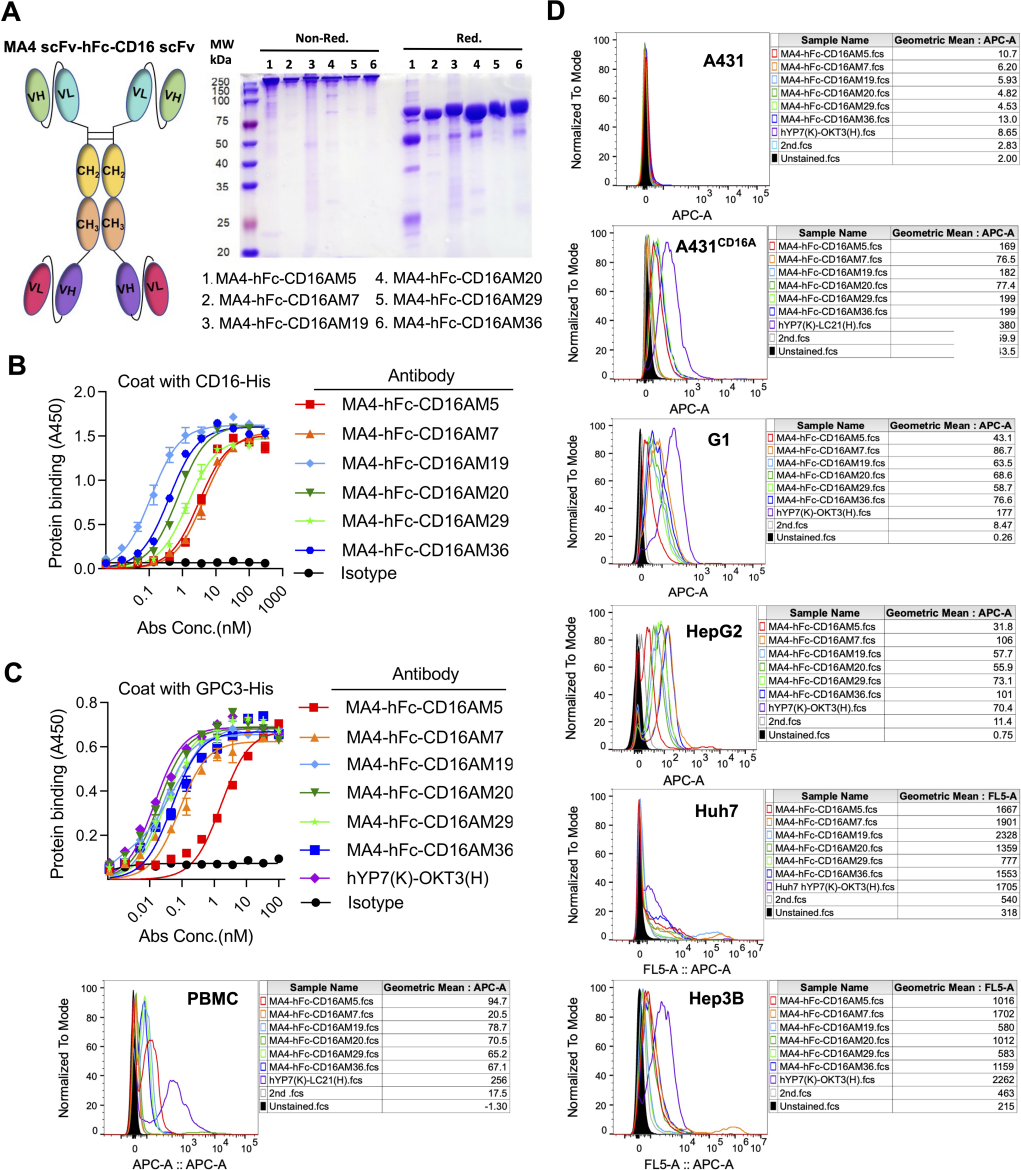
Design and *in vitro* characterization of CD16A/GPC3 bispecific antibody (BsAb). **(A)** Schematic of the scFv-hFc-scFv bispecific antibody (N297A Fc-silencing mutation) and SDS-PAGE validation (5 µg/lane). **(B, C)** ELISA-based binding kinetics of the BsAb against CD16A-His and GPC3-His. Plates coated with antigens (5 µg/mL) were incubated with BsAb (300 nM starting concentration, 3-fold serial dilutions). Binding was quantified using HRP-conjugated anti-human IgG. EC50 values were derived via GraphPad Prism. The GPC3/CD16-targeting BsAb hYP7(K)-LC21(H) served as a positive control. **(D)** Flow cytometric assessment of BsAb binding to CD16A-negative (A431) and CD16A-positive cell lines (A431-CD16A, Hep3B, HepG2, Huh7, PBMCs). Cells were incubated with MA4-hFc-CD16A (10 µg/mL, 90 min) and detected using Cy5-conjugated anti-human IgG.

A tandem scFv format was employed for bispecific antibody (BsAb) construction, linking GPC3-scFv-hFc(N297A) to CD16A-scFv (N297A mutation in Fc to eliminate glycosylation and ADCC; **Figure 2A**). BsAbs were expressed in 293F or CHO-S cells, purified via protein A affinity chromatography, and validated by SDS-PAGE. Under non-reducing conditions, the BsAbs formed ~300 kDa dimers composed of two 78 kDa single chains (**Figure 2A**). ELISA confirmed robust binding of MA4-hFc(N297A)-CD16A BsAbs (including M5, M7, M19, M20, M29, and M36) to both GPC3-His and CD16A-His (**Figures 2B, C**). Calculated EC50 values are summarized in **Table 2**.

**Table 2:** Summary of EC50 values for bispecific antibodies.

MA4-hFc(N297A)-CD16A		M5	M7	M19	M20	M29	M36
EC50 (nM)	GPC3	3.26	0.13	0.04	0.03	0.04	0.07
	CD16A	3.34	4.62	0.13	0.78	1.51	0.43

#### 3.2.2 Cellular targeting specificity

Flow cytometry assessed BsAb binding to A431 (CD16A-/GPC3-), GPC3+ hepatocellular carcinoma (HCC) lines (HepG2, Huh7, Hep3B), A431-GPC3 (G1), PBMCs, and A431-CD16A cells. Positive controls included hYP7(K)-OKT3(H) (T-cell targeting) and hYP7(K)-LC21(H).

(NK-cell targeting). At 10 μg/mL, all BsAbs bound specifically to GPC3+ HCC lines, G1 cells, and CD16A+ NK cells (comprising ~15% of PBMCs), but not to GPC3-/CD16A- A431 cells (**Figure 2D**). MA4-Fc(N297A)-CD16AM19 showed the strongest binding to Huh7, indicating recognition of native membrane-associated antigens.

Furthermore, fluorescence co-localization studies showed that in all cells examined, CD16AM5 was either free in the cytoplasm or bound to NK cells (**Supplementary Figure S1A**). This observation was also validated in A431 cells, where MA4-hFc-CD16AM5 showed a similar response. In the case of co-culture, the red fluorescence of Cy5 around tumor cells was observed to be in clear agreement with the cellular profile of PBMC (bright-field imaging) in multiple contact areas, especially in G1 and Huh7 cells. This finding demonstrates the efficacy of the BsAb in mediating spatial proximity and interaction between tumor cells and PBMCs.

### 3.3 *In vitro* cytotoxic effects

#### 3.3.1 BsAb-mediated live cancer cell cytotoxicity

**Figure 3:**
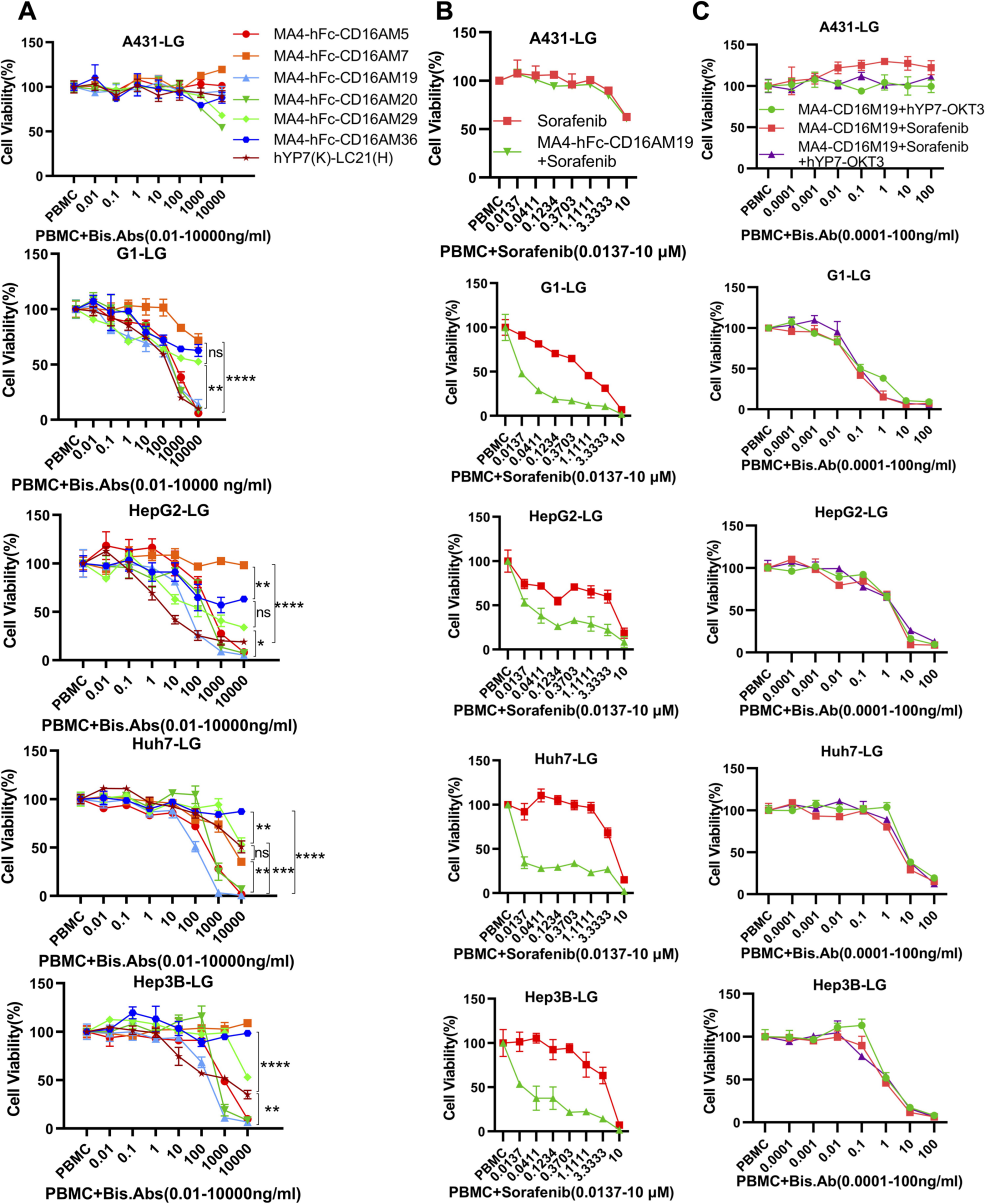
Dose-dependent cytotoxicity of CD16A/GPC3 BsAb and synergy with sorafenib. **(A)** Cytotoxic activity of BsAb against GPC3-negative (A431) and GPC3-positive tumor cells (A431-GPC3, HepG2, Huh7, Hep3B). Cells were co-cultured with PBMCs (20:1 ratio) and BsAb for 72 h. Viability was assessed via luciferase assay. Data represent mean ± SEM from two independent experiments. The GPC3/CD16-targeting BsAb hYP7(K)-LC21(H) was used as a positive control. **(B, C)** Synergistic cytotoxicity of MA4-hFc-CD16AM19 with sorafenib and hYP7(K)-OKT3(H). Experimental conditions matched panel **(A)**. Statistical significance determined by one-way ANOVA (****P < 0.0001). ns, Not Significant.

BsAb activity was evaluated in HCC lines and GPC3-A431 cells (negative control) using a luciferase-based cytotoxicity assay. All target cells were stably transduced with firefly luciferase. In co-culture models (E:T ratio = 20:1), nearly all BsAbs exhibited dose-dependent cytotoxicity against GPC3+ HepG2, Hep3B, Huh7, and G1 cells, with no effect on GPC3- A431 cells (**Figure 3A**). Among six BsAbs, MA4-Fc(N297A)-CD16AM5/19/20 demonstrated potent cytotoxicity against GPC3+ cells (particularly Huh7), with maximal killing observed at ~1,000 ng/mL for Hep3B, HepG2, and Huh7, and ~10,000 ng/mL for G1 cells (**Figure 3A**). IC50 values are detailed in **Table 3**. In contrast, MA4-Fc(N297A)-CD16AM7/29/36 showed significantly lower efficacy, particularly MA4-Fc(N297A)-CD16AM36. Subsequent experiments focused on MA4-Fc(N297A)-CD16AM5/19/20.

**Table 3:** The IC50 value of the bispecific antibody in relation to the elimination of GPC3-positive cells.

hYP7(K)-LC21(H)			M5/(ng/ml)	M7	M19	M20	M29	M36
IC50 (ng/ml)	A431	N/A						
	G1	136.4	453.3	N/A	202.9	266.2	N/A	N/A
	Huh7	N/A	295.5	N/A	92.75	574.9	N/A	N/A
	Hep3B	870.8	965.4	N/A	190.6	582.6	N/A	N/A
	HepG2	6.19	408.8	N/A	40.1	202.5	167.3	N/A

### 3.4 Sorafenib synergistic effects

MA4-hFc-CD16AM19 was prioritized for functional studies based on three criteria: (1) Highest binding avidity to both GPC3 (EC50 = 0.04 nM) and CD16A (EC50 = 0.13 nM) in ELISA (**Table 2**); (2) Optimal conformational epitope recognition confirmed by flow cytometry; (3) Superior thermal stability. Sorafenib, a multi-kinase inhibitor used in HCC therapy, was tested for synergy with MA4-hFc(N297A)-CD16AM19 (1 μg/mL). Dose-response curves shifted downward in combination groups (**Figure 3B**; IC50 in **Table 4**), indicating folds of enhancement in cytotoxicity at equivalent doses. In detail, for G1, HepG2, Huh7, and Hep3B, i.e. tumor cell lines, MA4-hFc-CD16AM1 in combination with sorafenib was significantly more effective in terms of cell killing than sorafenib alone (**Figure 3B**). For HepG2, the killing rate increased to 92.7 ± 3.8% (vs. 68.1 ± 4.5% for sorafenib alone; P < 0.01). Furthermore, we found that the combination of sorafenib, MA4-hFc(N297A)-CD16M19, and hYP7(K)-OKT3(H) also has a significant killing effect on liver cancer cells (**Figure 3C**). And the synergistic effect of the combination of the two or the three is better than that of the individual use (see **Supplementary Figure S1B**).

**Table 4:** The anti-tumor activity of M19 dual-antibody combined with sorafenib against tumor cells.

Cell line		Sorafenib only	Sorafenib +MA4-hFc(N297A)-CD16AM19
IC50 (ng/ml)	A431	N/A	N/A
	G1	0.75	0.02
	G2	2.23	0.03
	3B	3.4	0.03
	Huh7	5.23	0.01

### 3.5 *In vivo* antitumor efficacy

**Figure 4:**
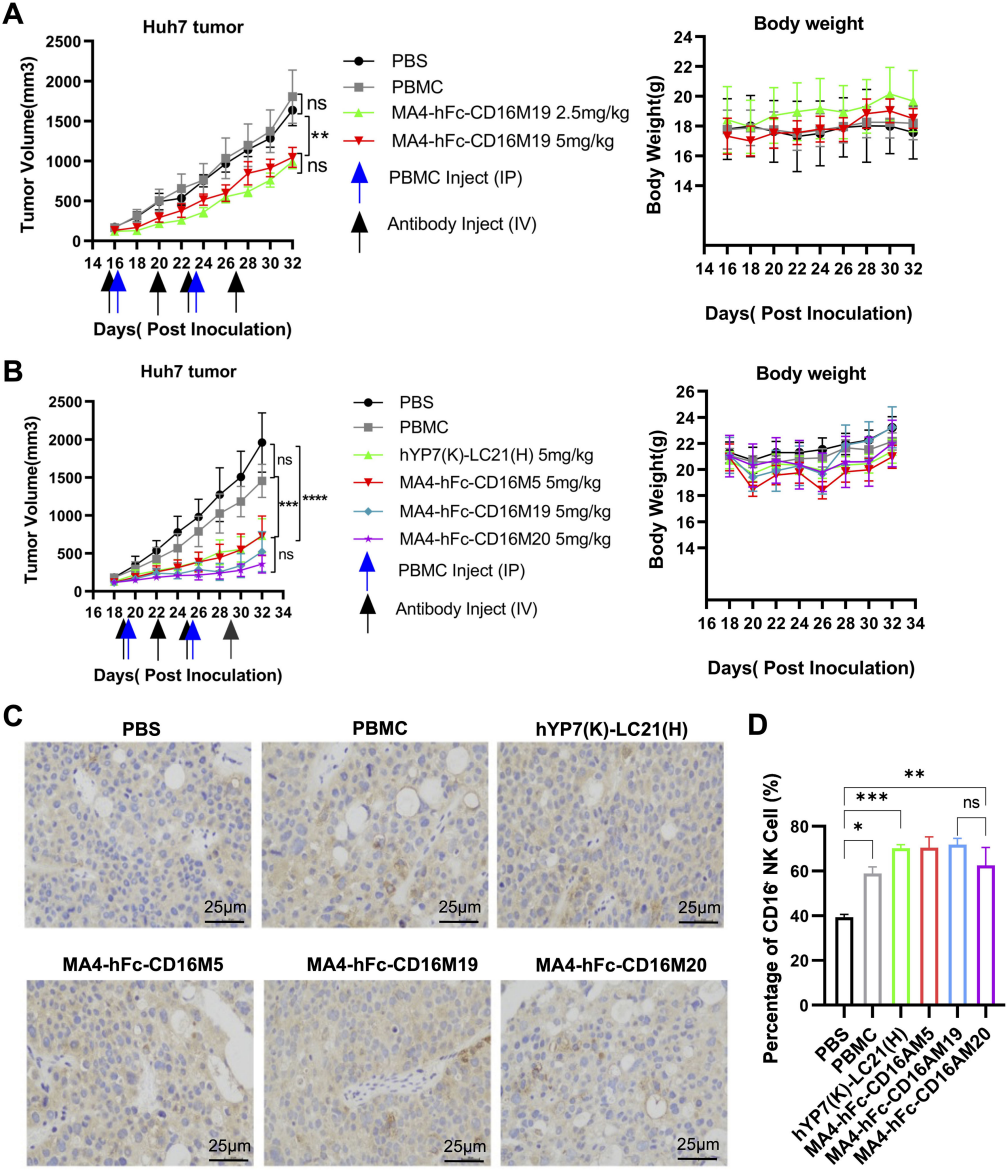
*In vivo* antitumor efficacy of lead BsAb correlates with NK cell infiltration. **(A, B)** Antitumor activity of MA4-hFc-CD16AM19 and comparator BsAbs in Huh7 xenograft models. NOD-SCID mice bearing 150 mm³ tumors (arrows) received weekly PBMC infusions (5 × 10^6^ cells) and biweekly BsAb doses (2.5–5 mg/kg, i.v.). Tumor volume (mean ± SEM, n = 5) and body weight were monitored. Statistical significance: **P < 0.01, ****P < 0.0001 (one-way ANOVA). **(C)** Immunohistochemical staining of tumor-infiltrating NK cells (CD56+). PBS-treated mice served as controls. Scale bar: 25 µm. **(D)** Quantification of NK cell infiltration. ***P < 0.001 (unpaired t-test). ns, Not Significant.

Huh7 xenograft models were established in NOD-SCID mice. Preliminary experiments with PBMCs (5 × 10^6^ cells/mouse, i.p.) and MA4-hFc(N297A)-CD16AM19 (2.5 or 5 mg/kg, i.v.) showed significant tumor growth inhibition without weight loss (**Figure 4A**). Subsequent studies compared MA4-hFc(N297A)-CD16AM5/19/20 and the positive control hYP7(K)-LC21(H). All BsAbs (5 mg/kg) suppressed tumor growth in Huh7 models (**Figure 4B**), with no adverse effects on body weight. Immunohistochemical staining of formalin-fixed, paraffin-embedded tumors revealed increased NK cell infiltration (CD16A+ cells) in BsAb-treated groups compared to controls (**Figure 4C**). Immunohistochemical (IHC) staining showed that the expression of the target protein was significantly higher in the experimental group than in the control group (**Figure 4D**).

## 4 Discussion

This study introduces a CD16A/GPC3 bispecific antibody (BsAb) platform as a transformative strategy for hepatocellular carcinoma (HCC) immunotherapy. Our CD16A/GPC3 BsAb platform addresses two unmet needs in HCC immunotherapy: (1) the lack of clinically advanced NK cell-engaging BsAbs and (2) the suboptimal synergy between existing BsAbs and TKIs. While T cell- redirecting BsAbs like ERY974 (GPC3/CD3) are under clinical evaluation, their reliance on T cells limits efficacy in the immunosuppressive HCC microenvironment. In contrast, NK cell-engagers like AFM13 (CD30/CD16A) have shown safety in hematologic malignancies but lack solid tumor targets [Rothe et al., 2015]. To our knowledge, MA4-hFc-CD16AM19 is the first CD16A/GPC3 BsAb optimized for HCC, combining species-specific NK cell activation with Fc engineering to minimize off-target effects. This aligns with recent advancements in BsAb design, where Fc-optimized formats improve tumor penetration and mitigate cytokine release syndrome (32).

### 4.1 Mechanistic advantages of CD16A/GPC3 BsAb design

The immunosuppressive HCC microenvironment often limits antibody-dependent cellular cytotoxicity (ADCC) due to CD16A polymorphism variability (e.g., 158V/F) and suboptimal NK cell activation (15, 34, 35). Our BsAb circumvents these limitations by directly engaging CD16A via its scFv domain, while the N297A mutation in the Fc region eliminates glycosylation-related off-target effects (e.g., thrombocytopenia) (36). This dual-targeting strategy strengthens immune synapse formation between NK and tumor cells. MA4-Fc(N297A)-CD16AM19 demonstrated superior cytotoxicity against GPC3+ HCC cells compared to traditional monoclonal antibodies (hYP7(K)-LC21(H)), consistent with Kontermann’s “dual-signal activation threshold” model, where optimal spatial geometry reduces antigen density requirements for NK activation (37). Flow cytometry and western blot analyses confirmed that MA4-Fc(N297A)-CD16AM19’s efficacy arises from its high affinity for native CD16A conformations, whereas antibodies targeting linear epitopes (e.g., CD16AM20) failed to recognize membrane-bound CD16A, underscoring the importance of epitope specificity (38, 39).

NK cell activation likely predominates due to CD16A’s abundant expression on NK cells (5–20% of lymphocytes) and low activation threshold (13). In contrast, T cell-engaging BsAbs (e.g., blinatumomab) require higher antigen densities and co-stimulatory signals, limiting their utility in solid tumors (40). Tumor histology revealed a 3.2-fold increase in CD56+CD16A+ NK cell infiltration in treated tumors, correlating with necrotic areas—a phenomenon analogous to CD16A/CD30 BsAb mechanisms in lymphoma (18, 19). The combination of MA4-hFc-CD16AM19 with sorafenib yielded significant synergy (CI=0.41), reducing the IC50 of sorafenib by 2.5- to 5.2-fold across HCC lines (**Table 4**). These findings align with Yang et al. (36), who proposed that kinase inhibitors enhance NK trafficking via vascular normalization. While vascular remodeling was not directly assessed, the observed increase in intratumoral CD16A+ NK cells (**Figure 4C**) supports improved immune cell infiltration, mirroring mechanisms reported for CD16A/CD30 BsAbs in lymphoma (19).

### 4.2 Challenges and future directions

Despite promising preclinical results, clinical translation requires addressing: 1) Target Heterogeneity: GPC3 expression varies (60–70% HCCs), necessitating companion diagnostics (e.g., 68Ga-GPC3 PET-CT) or multi-target strategies (e.g., GPC3/PD-L1 BsAbs). 2) Mechanistic Validation of Microenvironmental Modulation: While our data demonstrate synergy between MA4-hFc-CD16AM19 and sorafenib (CI=0.41) and enhanced NK cell infiltration (**Figure 4C**), direct interrogation of immune-suppressive pathways (e.g., TGF-β, IL-10, or checkpoint molecule dynamics) was beyond the current scope. Recent studies indicate that sorafenib remodels the TME by suppressing HIF-1α-driven TGF-β production, thereby alleviating NK cell suppression (41–43). Future work should quantify these cytokines in treated tumors and employ syngeneic HCC models to evaluate adaptive resistance mechanisms, such as compensatory PD-L1 upregulation or tumor-associated macrophage (TAM) recruitment. Combining CSF-1R inhibitors could mitigate this. 3) Pharmacokinetics: While the scFv-Fc design improved stability, its half-life (~50 h) remains shorter than IgG-based BsAbs (~200 h). FcRn-binding modifications or PEGylation may extend bioavailability. In summary, this BsAb platform represents a mechanistically innovative candidate for HCC immunotherapy. Future efforts should prioritize safety evaluations in non-human primates and biomarker-driven clinical trials to validate its therapeutic potential.

## 5 Conclusion

This study successfully developed a novel bispecific antibody (BsAb) platform targeting CD16A on NK cells and GPC3 in hepatocellular carcinoma (HCC), demonstrating three key innovations. First, species-specific CD16A engagement through high-affinity scFvs (EC50 <10 nM) circumvented polymorphism-related limitations while avoiding off-target FcγR activation via N297A Fc engineering. Second, the lead candidate MA4-hFc-CD16AM19 exhibited potent cytotoxicity against GPC3+ HCC cells (IC50 = 15–35 ng/mL) and synergized with sorafenib, reducing its IC50 by 2.5–5.2-fold through dual targeting of immune and kinase pathways. Third, *in vivo* validation confirmed significant tumor suppression without systemic toxicity, correlating with enhanced NK cell infiltration. These findings address critical barriers in HCC immunotherapy, including NK cell dysfunction and tumor microenvironment resistance. Future work should prioritize clinical translation through biomarker-driven patient stratification (e.g., GPC3 PET-CT) and combinatorial strategies targeting immunosuppressive factors like TGF-β or CSF-1R. By integrating Fc optimization, conformational epitope selection, and rational drug combinations, this BsAb platform represents a promising blueprint for next-generation solid tumor therapies.

## Data Availability

The raw data supporting the conclusions of this article will be made available by the authors, without undue reservation.
