# Supplementary figures and images for: Targeting CD16A on NK cells and GPC3 in hepatocellular carcinoma: development and functional validation of a therapeutic bispecific antibody
Source: Front Immunol. 2025 Jun 12;16:1599764. doi: 10.3389/fimmu.2025.1599764 (PMC12198245; doi:10.3389/fimmu.2025.1599764)

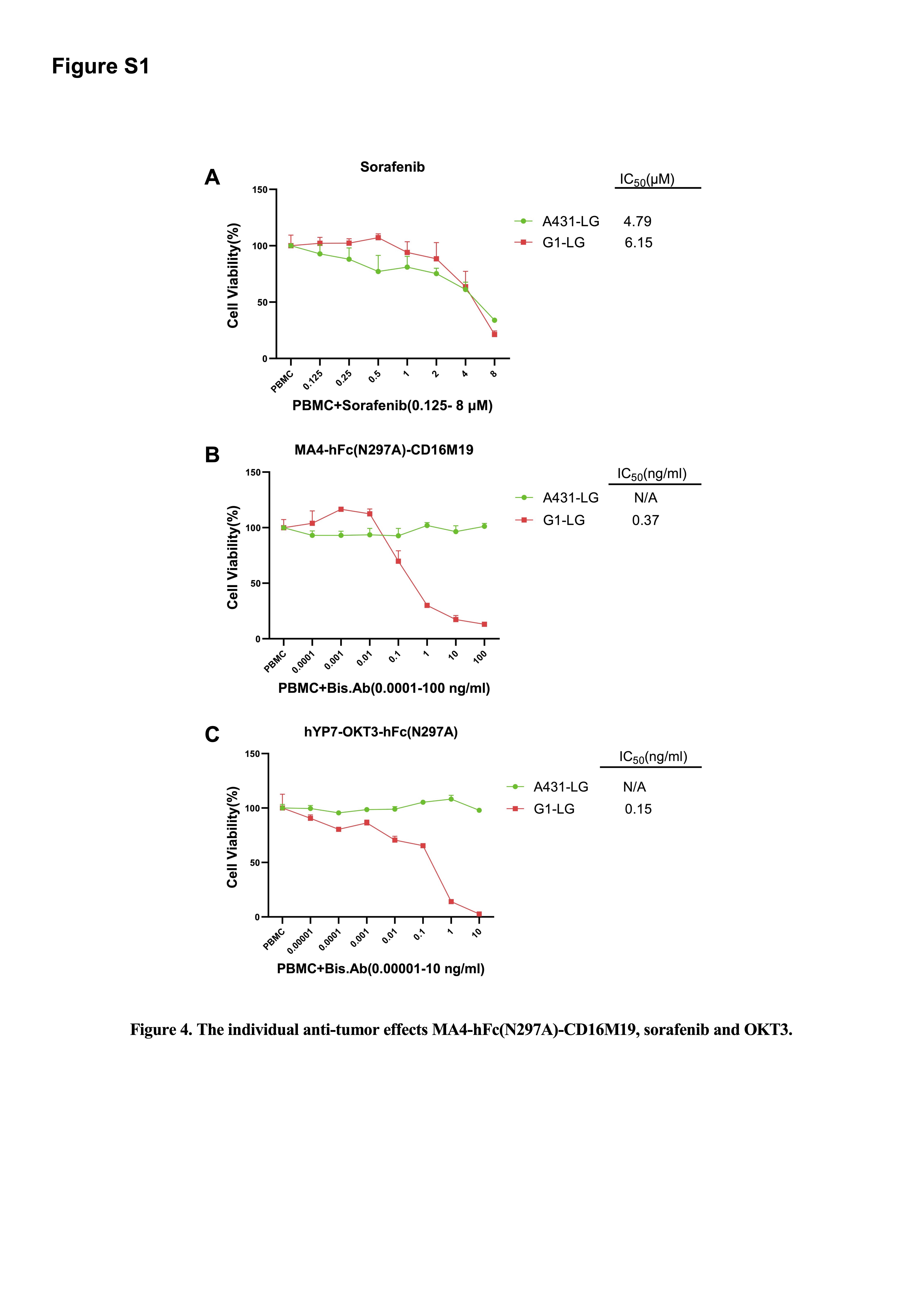

Supplement: Supplementary file 1 [file DataSheet1.zip › Supplement material/Figure S1.jpg]
